# Torsion of wandering spleen treated by laparoscopic splenopexy: A case report

**DOI:** 10.1016/j.ijscr.2019.06.040

**Published:** 2019-06-26

**Authors:** Mariyem Awan, Jose Luis Gallego, Annett Al Hamadi, Vijay Chander Vinod

**Affiliations:** a Department of Surgery, Mediclinic City Hospital, Dubai Healthcare City, Dubai, United Arab Emirates; b Department of Accident & Emergency, Mediclinic City Hospital, Dubai Healthcare City, Dubai, United Arab Emirates

**Keywords:** Case report, Wandering spleen, Laparoscopic splenopexy, Torsion of spleen, Acute abdomen

## Abstract

**Introduction:**

A wandering spleen is a mobile spleen as a result of deficient splenic peritoneal ligaments and elongation of its vascular pedicle. It is a rare entity affecting mainly young adults commonly females and children, presenting as an asymptomatic abdominal mass or abdominal discomfort due to torsion and de-torsion of the pedicle.

**Presentation of case:**

We report a 35-year-old female presented with intermittent colicky abdominal pain that worsened in severity over three weeks. Diagnostic laparoscopy showed torsion of spleen without infarction. Detorsion and Splenopexy in an extra-peritoneal pouch was performed. Post operatively, the patient recovered well and was healthy at two months follow up.

**Discussion:**

A wandering spleen is either congenital or acquired. The condition results in a long vascular pedicle, which predispose to the torsion resulting in a partial or complete infarct of the spleen. Laparoscopic approach is the preferred technique and de-torsion of the splenic pedicle and splenopexy is a reasonable surgical option, when there is no evidence of infarction of the spleen.

**Conclusion:**

The diagnosis of wandering spleen is very rare and extremely difficult to establish and is clinically nonspecific. An early diagnosis and surgical care are required for preserving the spleen. Additional imaging examinations can help establish a diagnosis.

## Highlights

• Wandering spleen.
• Laparoscopic splenopexy.
• Torsion of splenic pedicle.

## 1 Introduction

Wandering spleen is a rare clinical entity characterized by splenic hypermobility that results from the absence or maldevelopment of the splenic suspensory ligaments [1]. As a result, the spleen is predisposed to hilar torsion and subsequently infarction. The condition mainly affects the pediatric population in one third of cases [2]. In adults, females of reproductive age group are mostly affected, with the cause hypothesized to be hormonal changes during pregnancy leading to ligamentous laxity [2,3]. The symptoms are mainly because of torsion and spontaneous de-torsion of the spleen [4]. It is a diagnostic challenge due to the rarity of the condition. The management is planned according to the vitality of the organ at presentation. While wandering spleen has been traditionally treated by splenectomy, recently, splenic salvage by untwisting of the hilar vessels and subsequent splenopexy is being increasingly advocated particularly in pediatric population. This case report presents a rare case of wandering spleen that was successfully treated by laparoscopic detorsion and splenopexy in an extra-peritoneal pouch. The work has been reported in line with the SCARE criteria [5].

## 2 Presentation of case

A 35-year-old lady, presented thrice to the emergency department within a week with the complaints of severe intermittent colicky abdominal pain, increasing in intensity upon admission. No associated symptoms such as vomiting, fever and constipation. She complained of similar episodes of intermittent mild pain since three years. She had a past history of ovarian cystectomy and LSCS twice. She smokes 40 cigarettes per day, does not consume alcohol. She has contrast allergy.

On general examination, the patient was afebrile (37 °C), pulse rate of 90 bpm, and blood pressure of 120/70 mmHg. No pallor, jaundice, finger clubbing or lymphadenopathy noticed. Systemic examination was unremarkable. An abdominal examination revealed mild tenderness and no guarding in the left hypochondrium.

Laboratory parameters showed Hemoglobin 12.3 gm/dl (11.5–15.5), white blood cells 7.0 K/μL (4.011.0) and the platelet count was 200.0 K/μL (150.0–450.0); CRP 5 mg/L (0.0–5.0), ESR 18 mm/h (0–32), serum amylase 58 IU/L (25–125), lipase 30 U/L (8–78). LFTs, urea and electrolytes were normal.

**Fig. 1:**
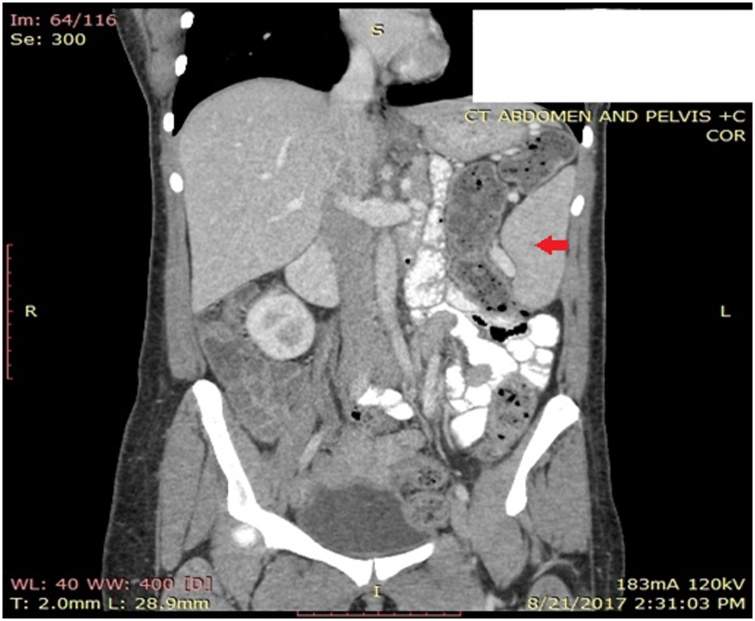
Spleen.

**Fig. 2:**
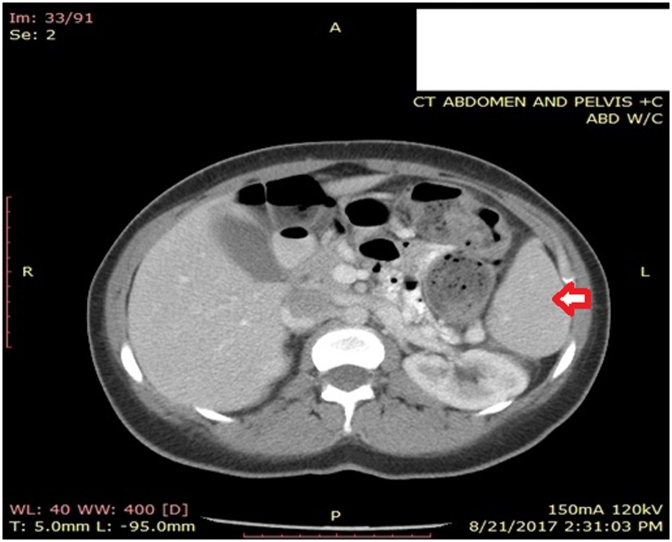
Spleen.

A computed tomography (CT) scan of the abdomen with oral and IV contrast was performed. As shown in Fig. 1, Fig. 2 an anterior displacement of the spleen and splenic vascular pedicle was noticed. The splenic pedicle was rotated and formed a tornado shape structure that was associated with marked regional venous dilatation, suggestive of wandering spleen. This displacement of the spleen was found to be due to defect of ligamentous attachments of the spleen. The stomach was indented by the displaced spleen and the tail of pancreas was located in sub phrenic position. There was no evidence of regional fat stranding and of arterial obstruction or spleen structure abnormality. Likewise, there was no evidence of free intra abdominal fluid or collection but the proximal intestinal loop showed moderate wall thickness and food residue. The cecum was filled with fecal material and was lying in lower abdomen and appendix located in left lower abdomen. A right ovarian follicular cyst was noted measuring 2.5 × 2 cm.

**Fig. 3:**
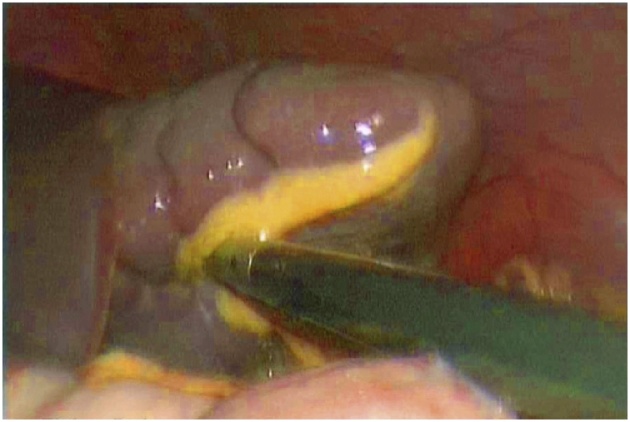
Spleen.

**Fig. 4:**
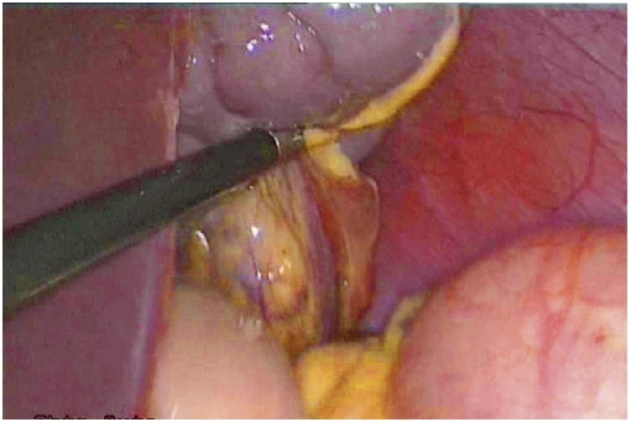
Spleen.

**Fig. 5:**
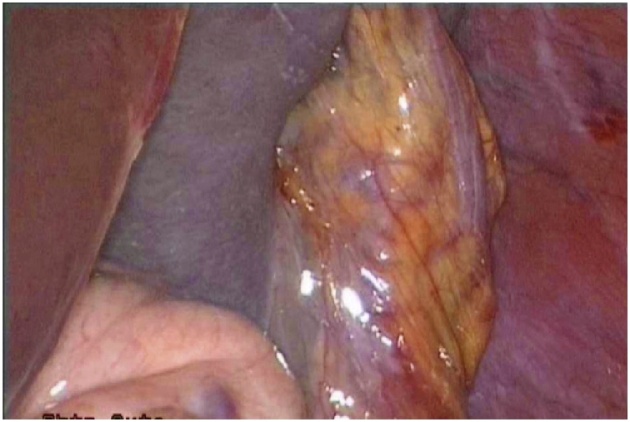
Spleen.

**Fig. 6:**
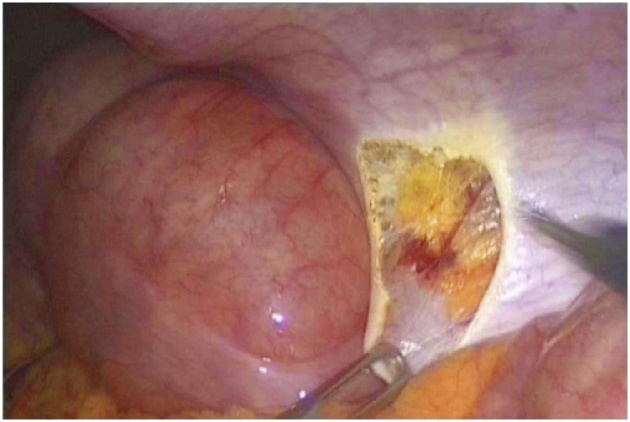
Spleen.

**Fig. 7:**
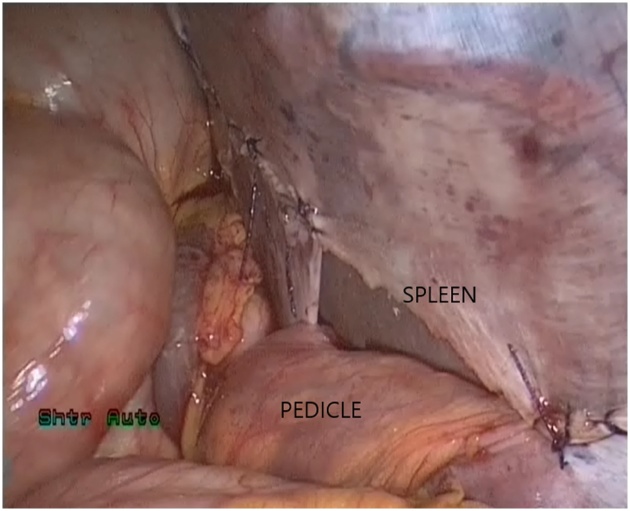
Spleen.

The patient was scheduled for a diagnostic laparoscopy under general anesthesia. The operative findings showed that showed an enlarged and congested spleen overlying the bowels (Fig. 3, Fig. 4) and the absence of the spleen from its normal position. All of the splenic ligamentous attachments, gastrosplenic, colicosplenic, phrenocolic and splenorenal ligaments, were completely absent. The adhesions between the spleen and transverse colon and omentum were released by using a Harmonic scalpel (Ethicon, Cincinnati, OH). The spleen was not infracted but was congested and twisted three times around its long vascular pedicle (Fig. 5). The torsion of the splenic pedicle was untwisted in a counterclockwise direction. Upon untwisting of the pedicle, the splenic congestion and its size reduced dramatically. The posterior peritoneum over the left kidney was opened, and a flap including peritoneum over the anterior abdominal wall was lifted up. The extra peritoneal pouch was created in the left lateral abdominal wall just below the 11th rib (Fig. 6). Next, the spleen was placed in between the extra peritoneal pouch and the splenopexy was performed (Fig. 7). The open ends of the peritoneum were sutured with V-Lock absorbable sutures, thus preventing the organ from slipping out. Post-operatively, the patient had uneventful recovery. Oral liquids were allowed on the first

2^nd^ postoperative day and the patient consumed a normal diet on the post-operative day. Thereafter, she was discharged on oral analgesia. She was followed up for two months post operatively without any sinister complaints.

## 3 Discussion

A wandering spleen is either congenital or acquired [6,7]. The congenital anomalies, such as hypermobile colon and Prune Belly Syndrome, are associated with this disease [8]. The acquired anomalies usually occur in reproductive age of young women, commonly multiparous, which suggests that pregnancy may contribute to ligamentous lengthening due to laxity of the abdominal wall and hormonal changes [4]. The condition results in a long vascular pedicle, which predispose to the torsion resulting in a partial or complete infarct of the spleen.

Sonography may show characteristic comma-shaped spleen in an ectopic position and the lack of splenic tissue in the left upper quadrant. Multi slice spiral CT scan is the investigation of choice to demonstrate the position and the viability of spleen [9,10]. Preservation of spleen is highly recommended in young patients. Laparoscopic approach is the preferred technique and de-torsion of the splenic pedicle and splenopexy is a reasonable surgical option, when there is no evidence of infarction of the spleen [11,12].

The various techniques of splenopexy have been described in the literature such as splenopexy in an extra peritoneal pouch [13] or creating a pouch in the omentum, stomach, or colon and the use of absorbable mesh to fix the spleen in its normal anatomical location [14]. Both procedures have achieved comparable outcomes with acceptable patients’ compliance.

## 4 Conclusion

The diagnosis of wandering spleen is very rare and extremely difficult to establish and is clinically nonspecific. An early diagnosis and surgical care are required for preserving the spleen. Additional imaging examinations can help establish a diagnosis. Laparoscopy usually confirms the diagnosis. When wandering spleen is diagnosed, splenopexy is the treatment of choice, irrespective of the presence or absence of symptoms, even without the presence of splenic infarction and necrosis. Splenopexy is feasible, less invasive, and does not affect the splenic function. If splenic necrosis is present, splenectomy usually is required.

## Funding

No funding.

## Ethical approval

Ethical approval taken from the Mediclinic Research & Ethic Committee.

## Consent

The head of our medical team/hospital or legal team have taken responsibility that exhaustive attempts have been made to contact the family and that the paper has been sufficiently anonymised not to cause harm to the patient or their family. A signed document to this effect can be provided on request.

## Author contribution

**Dr. Mariyem Awan** – Original Draft writing, editing and literature review.

**Dr. Jose Luis Gallego** – Laparoscopic Surgeon who performed Splenopexy.

**Dr. Annett Al Hamadi** – Reviewing the draft.

**Dr. Vijay Chander Vinod** – Emergency Physician who 1^st^ saw the patient and performed the CT to establish initial diagnosis. Draft writing and editing.

## Registration of research studies

Not applicable.

## Guarantor

Dr Mariyem Awan.

Dr Vijay Vinod.

## Provenance and peer review

Not commissioned, externally peer-reviewed.

## Declaration of Competing Interest

No conflicts of interest.
